# Exploration of neuroanatomical characteristics to differentiate prodromal Alzheimer’s disease from cognitively unimpaired amyloid-positive individuals

**DOI:** 10.1038/s41598-024-60843-8

**Published:** 2024-05-02

**Authors:** Hak Hyeon Kim, Min Jeong Kwon, Sungman Jo, Ji Eun Park, Ji Won Kim, Jae Hyoung Kim, Sang Eun Kim, Ki Woong Kim, Ji Won Han

**Affiliations:** 1 Department of Neuropsychiatry, Seoul National University Bundang Hospital, 82 Gumi-ro 173 Beon-gil, Bundang-gu, Seongnam-si, Gyeonggi-do 13620 South Korea; 2 Department of Psychiatry, College of Medicine, Seoul National University, Seoul, South Korea; 3 Department of Brain and Cognitive Science, College of Natural Sciences, Seoul National University, Seoul, South Korea; 4 Department of Health Science and Technology, Graduate School of Convergence Science and Technology, Seoul National University, Seoul, South Korea; 5 Department of Radiology, Seoul National University Bundang Hospital, College of Medicine, Seoul National University, Seongnam-si, Gyeonggi-do South Korea; 6 Department of Nuclear Medicine, Seoul National University Bundang Hospital, College of Medicine, Seoul National University, Seongnam-si, Gyeonggi-do Korea; 7 Center for Nanomolecular Imaging and Innovative Drug Development, Advanced Institutes of Convergence Technology, Suwon, Republic of Korea

**Keywords:** Alzheimer's disease, Learning and memory

## Abstract

Differentiating clinical stages based solely on positive findings from amyloid PET is challenging. We aimed to investigate the neuroanatomical characteristics at the whole-brain level that differentiate prodromal Alzheimer’s disease (AD) from cognitively unimpaired amyloid-positive individuals (CU A+) in relation to amyloid deposition and regional atrophy. We included 45 CU A+ participants and 135 participants with amyloid-positive prodromal AD matched 1:3 by age, sex, and education. All participants underwent ^18^F-florbetaben positron emission tomography and 3D structural T1-weighted magnetic resonance imaging. We compared the standardized uptake value ratios (SUVRs) and volumes in 80 regions of interest (ROIs) between CU A+ and prodromal AD groups using independent t-tests, and employed the least absolute selection and shrinkage operator (LASSO) logistic regression model to identify ROIs associated with prodromal AD in relation to amyloid deposition, regional atrophy, and their interaction. After applying False Discovery Rate correction at < 0.1, there were no differences in global and regional SUVR between CU A+ and prodromal AD groups. Regional volume differences between the two groups were observed in the amygdala, hippocampus, entorhinal cortex, insula, parahippocampal gyrus, and inferior temporal and parietal cortices. LASSO logistic regression model showed significant associations between prodromal AD and atrophy in the entorhinal cortex, inferior parietal cortex, both amygdalae, and left hippocampus. The mean SUVR in the right superior parietal cortex (beta coefficient = 0.0172) and its interaction with the regional volume (0.0672) were also selected in the LASSO model. The mean SUVR in the right superior parietal cortex was associated with an increased likelihood of prodromal AD (Odds ratio [OR] 1.602, p = 0.014), particularly in participants with lower regional volume (OR 3.389, p < 0.001). Only regional volume differences, not amyloid deposition, were observed between CU A+ and prodromal AD. The reduced volume in the superior parietal cortex may play a significant role in the progression to prodromal AD through its interaction with amyloid deposition in that region.

## Introduction

Alzheimer’s disease (AD), the most prevalent form of dementia^1,2^, begins with a preclinical stage representing a continuum of asymptomatic individuals with biomarker evidence, such as the accumulation of beta-amyloid (Aβ) plaques^3^. This phase is followed by prodromal AD, in which individuals experience cognitive impairment short of dementia, with an impaired/abnormal range in objective cognitive tests^4^. In other words, both preclinical and prodromal AD require amyloid positivity, but are distinguished by the presence of cognitive impairment, representing cognitively normal and mild cognitive impairment (MCI), respectively. Several studies have investigated the neuroanatomical progression of amyloid deposition to understand the progression of these clinical stages in the AD continuum^5–7^.

Based on regional amyloid positron emission tomography (PET), multiple studies have developed and implemented models for staging amyloid pathology^8^. Compared to Stage 0 with no amyloid, participants with MCI may correspond to amyloid deposition in the precuneus, posterior and isthmus cingulate, insula, orbitofrontal, medial/lateral frontal, lateral parietal, temporal, and occipital cortices (neocortex)^6,9–11^. Recent studies, which estimated a regional progression pattern of amyloid deposition from cross-sectional^7^ or longitudinal^12,13^ amyloid PET data, reported that amyloid deposition begins in the temporobasal and frontomedial areas (Stage I) and successively affects the remaining associative neocortex (Stage II), primary sensory-motor areas and the medial temporal lobe (Stage III), and finally the striatum (Stage IV). Although clinical stages could not exactly match the specific amyloid stage, MCI showed an increased proportion of Stages III–IV compared to cognitively normal individuals^7,12^. However, these amyloid PET staging studies usually include amyloid-negative cognitively normal participants as part of the preclinical AD, making it challenging to identify regions associated with progression from preclinical to prodromal stages within the amyloid-positive AD continuum.

Amyloid deposition does not account for all progression of AD^14^; several longitudinal studies^15–20^ have reported that a substantial number of CU A+ individuals remained cognitively unimpaired over the years. However, those who did progress showed higher baseline 18F-Florbetapir uptake^17^ and both amyloid and tau pathologies in cognitively unimpaired individuals elevate the risk of progressing to prodromal AD^19,20^. Therefore, additional downstream biomarkers are important for disease progression. In particular, tau pathology and region-specific atrophy play crucial roles in the interpretation of the clinical manifestation of AD^21,22^. Moreover, the interactive or synergistic effects of amyloid burden and neurodegeneration may also affect the progression of AD in Aβ-associated cortical regions (right lateral temporo-parietal, right precuneus, right superior parietal, right caudal middle frontal, or left lateral occipital regions^23^) or AD-vulnerable regions (lateral parietal, lateral inferior temporal, and posterior cingulate cortices)^24^. However, these studies^23,24^ dichotomized amyloid deposition in positive/negative, which limited the investigation of the quantitative influence of amyloid that may interact with neurodegeneration. Another limitation of these studies is that they explored interactive effects only within regions associated with amyloid rather than across the entire brain region.

In this study, we explored the neuroanatomical characteristics that may differentiate prodromal AD from cognitively unimpaired amyloid-positive individuals (CU A+) by comparing regional amyloid deposition and volume between these stages throughout the cortex and subcortex. To explore the pivotal regions associated with the prodromal AD stage compared to CU A+, we identified specific regions that show significant interactions between amyloid deposition and regional atrophy in relation to progression to prodromal AD.

## Methods

### Participants

We recruited participants from the Korean Longitudinal Study on Cognitive Aging and Dementia (KLOSCAD) and visitors to the dementia clinic of Seoul National University Bundang Hospital (SNUBH) between August 2016 and August 2022. KLOSCAD is a nationwide, prospective cohort study that included a random sample of 6818 Koreans aged ≥ 60 years. The initial assessment was carried out between 2010 and 2012 with biennial follow-ups^25^. We included a total of 180 participants, 45 participants with CU A+ and 135 with prodromal AD matched 1:3 by age, sex, and education. All participants were amyloid-positive, as confirmed by a global standardized uptake value ratio (SUVR) of ≥ 0.96^26^ using ^18^F-florbetaben PET scans. We excluded participants with major psychiatric and/or neurological disorders other than AD, which could influence cognitive function. This study was approved by the Institutional Review Board of the SNUBH, Seongnam, Korea. We acquired written informed consent from the subjects or their legal guardians. All procedures were performed in accordance with the relevant guidelines and regulations.

### Diagnostic evaluation

Geriatric neuropsychiatrists conducted standardized diagnostic interviews that included medical history evaluation, physical examination, and neurological assessment according to the Korean version of the Consortium to Establish a Registry for AD Assessment Packet Clinical Assessment Battery (CERAD-K)^27^. Research neuropsychologists or trained research nurses administered the Korean version of the Consortium to Establish a Registry for Alzheimer’s Disease Assessment Packet Neuropsychological Assessment Battery (CERAD-K-N)^28,29^, digit span test (DST)^30^, executive clock drawing task (CLOX)^31,32^, and frontal assessment battery^33^ to each participant. The CERAD-K-N consists of nine neuropsychological tests: verbal fluency test, 15-item Boston naming test, mini-mental state examination, word list memory test, constructional praxis test, word list recall test, word list recognition test, constructional recall test, and trail making test A/B^28,29^.

A panel of geriatric neuropsychiatrists determined the final diagnosis and clinical dementia rating (CDR)^34^. Cognitive stages were determined by syndrome staging of the cognitive continuum at the National Institute on Aging and Alzheimer’s Association (NIA-AA)^4^. CU A + was defined as cognitively unimpaired individuals with a CDR = 0 or neuropsychological performance greater than − 1.5 standard deviations (SD) from the age-, sex-, and education-adjusted norms in all neuropsychological tests. Prodromal AD was identified as MCI when the subject’s performance was less than − 1.5 SD of age-, sex-, and education-adjusted norms in any neuropsychological test.

### Acquisition of brain magnetic resonance imaging (MRI) and regional volume

Three-dimensional T1-weighted spoiled gradient-echo MRI was performed using a 3.0 T Achieva Scanner (Philips Medical Systems, Eindhoven, the Netherlands) at SNUBH. The images featured a sagittal slice thickness of 1.0 mm with no gaps between slices, an echo time of 4.6 ms, a repetition time of 8.1 ms, a flip angle of 8°, and a matrix size measuring 175 × 480 × 480 in the x, y, and z dimensions, with a voxel size of 1.0 × 0.5 × 0.5 mm^3^. We converted the original digital imaging and communications in medicine format images to the neuroimaging informatics technology initiative format for analysis using MRIcron software (version 1.0; https://www.micro.com). Subsequently, we resliced the T1 images to isovoxels sized 1.0 × 1.0 × 1.0 mm^3^.

We used FreeSurfer (version 6.0.0; http://surfer.nmr.mgh.harvard.edu) to segment whole-brain structures into brain regions as defined by the Desikan–Killiany–Tourville (DKT) atlas^35^. The process begins with motion correction, nonuniform intensity normalization, and skull stripping. The second stage involves full-scale volumetric labeling and automatic topology fixing. The third stage comprises spherical mapping and cortical parcellation. After completing the recon-all process, we obtained individual parcellated brain masks according to the DKT atlas^36^. We extracted regional volumes of 68 regions of interest (ROIs) of the cerebral cortex^37^ and 12 ROIs of the subcortex^36^.

### Acquisition of ^18^F-florbetaben brain PET scans and regional SUVRs

Amyloid brain PET images with ^18^F-florbetaben were performed with a discovery VCT scanner (General Electric Medical Systems; Milwaukee, WI, USA). An intravenous slow bolus injection (6 s/mL) of 8.1 mCi (300 MBq) was administered. ^18^F-florbetaben (Neuraceq, Piramal, Mumbai, India) was administered in a total volume of up to 10 mL. After a 90-min uptake period, 20-min PET images were captured, consisting of four 5-min dynamic frames.

Images were processed using the FreeSurfer PetSurfer procedure (FreeSurfer version 6.0.0; http://surfer.nmr.mgh.harvard.edu/fswikiPetSurfer/) to perform co-registration, SUVR calculations, and partial volume effect correction (PVC)^38^. In detail, the individual PET was co-registered with the corresponding native T1‐weighted MRI using a rigid‐body registration with a mutual information cost function. Each individually co-registered PET scan was then scaled by the mean value in the cerebellar reference region to calculate the SUVR. The global SUVR was calculated as the volume-average uptake of five cortical ROIs, including the frontal, parietal, lateral temporal, posterior, and anterior cingulate cortex regions, to the whole-cerebellar reference region^39^. The amyloid‐positivity was defined considering the threshold of global SUVR ≥ 0.96 in each individual^26^ in this study. Individual SUVR images were corrected for partial volume effects using an extended Müller–Gärtner (MG) method, which estimates the true radioactivity concentration in the gray matter (GM) of the human brain by considering the heterogeneity of the GM activity through a four-compartment model within the PetSurfer procedure. The GM threshold for PVC was set to 0.1, and the point spread function for PVC was estimated to be 4 mm. We computed regional SUVR values from ROIs used for regional volume estimation by MRI using PetSurfer procedure.

### Statistical analysis

We compared demographic and clinical characteristics between the two diagnostic groups (CU A+ and prodromal AD groups) using the independent t-test (continuous variables) and chi-square test (categorical variables). We compared the regional SUVR and volume adjusted for intracranial volume (ICV) across all ROIs in the DKT atlas between the two groups using independent t-tests. Subsequent multiple comparisons were conducted using the False Discovery Rate (FDR), establishing a significance threshold for group differences at < 0.1^40–43^. Furthermore, we compared the regional mean SUVR in each ROI using a two-way analysis of covariance adjusted for the volume of each ROI.

We then used LASSO logistic regression to identify ROIs associated with prodromal AD in relation to amyloid deposition and regional atrophy. LASSO regression is a technique that emphasizes variables with robust associations with the outcome of interest while identifying the coefficients that should be set to zero, effectively facilitating feature selection and reducing model complexity^44^. All continuous predictor variables, including SUVR and volume values for each ROI, were standardized before fitting the model to ensure comparability and improve the interpretability of the model coefficients. To assess the uncertainty of the model coefficients, we performed a bootstrap analysis with 1000 replications. This approach allowed us to estimate the standard errors and 95% confidence intervals (Cis) for the LASSO logistic regression coefficients by resampling the data with replacement and refitting the model for each replication^45^. We tested three different LASSO logistic regression models: (1) the model included SUVR values for all ROIs; (2) the model included regional volumes of all ROIs; and (3) the model included SUVR and volume of each ROI, along with interaction terms, to explore the interplay between regional amyloid deposition and regional atrophy.

In ROIs that show a significant interaction between regional SUVR and volume, we performed a subgroup analysis. We divided the entire group into high and low SUVR groups according to the median value of SUVR in the respective ROI and analyzed the association between SUVR levels and prodromal AD using binary logistic regression. Similarly, we divided the entire group into the high- and low-volume groups according to the median value of volume in the respective ROI and analyzed the association between volume and prodromal AD.

Statistical significance was determined by a two-tailed p-value of less than 0.05 for all analyses. The Statistical Package for the Social Sciences version 25.0 (IBM Corporation; Armonk, NY, USA) and R version 4.2.2 (The R Foundation for Statistical Computing; Vienna, Austria) were used for all statistical analyses.

## Results

The mean age of the 180 participants was 76.82 years; 60% were female, and the mean educational level was 12.24 years. In the prodromal AD group, the proportion of apolipoprotein E4 carriers was higher and most neuropsychological test scores were lower than in the CU A+ group; however, these differences were not statistically significant. However, in categorical verbal fluency, constructional praxis, and wordlist recognition tests, the prodromal AD group showed significantly worse performance than the CU A+ group. Although the global SUVR did not differ significantly between the two groups, the total GM volume in the prodromal AD group was significantly lower than in the CU A+ group (Table 1).

**Table 1:** Demographic and clinical characteristics of the participants.

Characteristics	All	CU A+	Prodromal AD	p-value*
Number of subjects	180	45	135	
Age (SD), years	76.82 (5.37)	76.56 (5.36)	76.91 (5.39)	0.702
Sex				1.000
Female, n (%)	108 (60%)	27 (60%)	81 (60%)	
Male, n (%)	72 (40%)	18 (40%)	54 (40%)	
Education (SD), y	12.24 (4.65)	12.51 (4.79)	12.16 (4.61)	0.658
APOE ε4 carrier				0.070
APOE ε4−, n (%)	83 (46.1%)	26 (57.8%)	57 (42.2%)	
APOE ε4+, n (%)	97 (53.9%)	19 (42.2%)	78 (57.8%)	
Verbal fluency (SD)^a^	12.90 (4.52)	16.22 (4.92)	11.72 (3.74)	0.038
Boston naming test (SD)^a^	10.80 (2.60)	11.93 (2.05)	10.40 (2.66)	0.108
MMSE (SD)	24.31 (3.75)	26.38 (2.77)	23.61 (3.78)	0.086
Construction praxis test (SD)^a^	9.86 (1.48)	10.18 (1.01)	9.75 (1.60)	0.043
Word list memory (SD)^a^	13.66 (4.22)	17.62 (3.80)	12.25 (3.40)	0.596
Word list recall (SD)^a^	2.49 (2.49)	5.22 (2.04)	1.53 (1.84)	0.194
Word list recognition (SD)^a^	6.71 (2.82)	9.20 (1.22)	5.83 (2.70)	< 0.001
Construction recall test (SD)^a^	3.67 (3.18)	6.60 (2.62)	2.63 (2.67)	0.671
Trail making test A (SD)^a^	71.37 (48.00)	58.22 (33.06)	76.03 (51.89)	0.055
Trail making test B (SD)^b^	227.9 (102.38)	181.48 (100.37)	244.66 (98.22)	0.555
Digit span forward (SD)^c^	5.73 (1.27)	5.93 (1.20)	5.65 (1.30)	0.644
Digit span backward (SD)^c^	0.44 (1.19)	0.56 (0.99)	0.39 (1.26)	0.159
Frontal assessment battery (SD)^c^	14.36 (2.57)	15.22 (2.22)	14.05 (2.63)	0.578
Global SUVR (SD, range)	1.41 (0.23, 0.98–2.18)	1.37 (0.22, 1.02–1.89)	1.42 (0.23, 0.98–2.18)	0.231
Total GMV/ICV (SD)	0.35 (0.02)	0.36 (0.03)	0.35 (0.02)	0.006

*AD* Alzheimer’s disease, *APOE* apolipoprotein E, *CU A*+ cognitively unimpaired amyloid-positive individual, *GMV* gray matter volume, *ICV* intracranial volume, *MMSE* Mini-Mental State Examination, *SD* standard deviation, *SUVR* standardized uptake value ratio.

*Independent t-tests for continuous variables, Chi-square tests for categorical variables.

^a^Missing data n = 3.

^b^Missing data n = 9.

^c^Missing data n = 4.

After adjusting for multiple comparisons, we found no significant differences in SUVR values across the 80 ROIs in the DKT atlas between the two groups (Table 2, Supplementary Table S1, Fig. S2). However, significant differences in regional volumes were observed in 10 ROIs across the groups: two ROIs in the limbic area (right insula and right parahippocampal gyrus), three ROIs in the temporal lobe (both entorhinal cortices and left inferior temporal gyrus), one ROI in the parietal lobe (right inferior parietal cortex), and four ROIs in the subcortex (both amygdalae and both hippocampi).

**Table 2:** Comparisons of regional SUVR and volume between cognitively unimpaired amyloid-positive individual and prodromal Alzheimer’s disease.

Regions of interest*	SUVR			Regional volume/ICV (%)		
	CU A+	Prodromal AD	p-value	CU A+	Prodromal AD	p-value
Limbic system						
Right insula	1.04 ± 0.18	1.06 ± 0.22	0.508	0.43 ± 0.04	0.41 ± 0.04	0.003^a,b^
Right parahippocampal gyrus	0.96 ± 0.19	0.98 ± 0.23	0.491	0.11 ± 0.02	0.10 ± 0.02	0.002^a,b^
Temporal lobe						
Left entorhinal cortex	0.67 ± 0.18	0.64 ± 0.23	0.374	0.13 ± 0.02	0.10 ± 0.03	< 0.001^a,b^
Left inferior temporal gyrus	1.34 ± 0.29	1.34 ± 0.31	0.984	0.64 ± 0.08	0.60 ± 0.08	0.009^a,b^
Right entorhinal cortex	0.67 ± 0.17	0.67 ± 0.21	0.939	0.12 ± 0.02	0.11 ± 0.02	< 0.001^a,b^
Parietal lobe						
Right inferior parietal cortex	1.50 ± 0.26	1.57 ± 0.30	0.138	0.81 ± 0.09	0.77 ± 0.10	0.006^a,b^
Subcortex						
Left amygdala	1.30 ± 0.27	1.33 ± 0.26	0.584	0.08 ± 0.01	0.07 ± 0.01	< 0.001^a,b^
Left hippocampus	1.44 ± 0.36	1.49 ± 0.36	0.465	0.22 ± 0.03	0.20 ± 0.03	< 0.001^a,b^
Right amygdala	1.32 ± 0.25	1.35 ± 0.25	0.442	0.09 ± 0.01	0.09 ± 0.01	< 0.001^a,b^
Right hippocampus	1.46 ± 0.24	1.45 ± 0.30	0.788	0.23 ± 0.04	0.21 ± 0.03	< 0.001^a,b^

*AD* Alzheimer’s disease, *CU A*+ cognitively unimpaired amyloid-positive individual, *ICV* intracranial volume, *ROI* region of interest, *SD* standard deviation, *SUVR* standardized uptake value ratio.

*Only ROIs from the Desikan–Killiany–Tourville atlas showing significant differences in regional volume or SUVR between the two groups are presented in this table. The comparison results for all ROIs can be found in the Supplementary Table S1.

*Standardized data are presented as mean ± SD.

^a^p < 0.05 from independent t-test between CU A + and prodromal AD.

^b^False Discovery Rate adjusted p value < 0.1 from independent t-test between CU A+ and prodromal AD; no regions of interest were significant at FDR p < 0.05 level.

**Figure 1:**
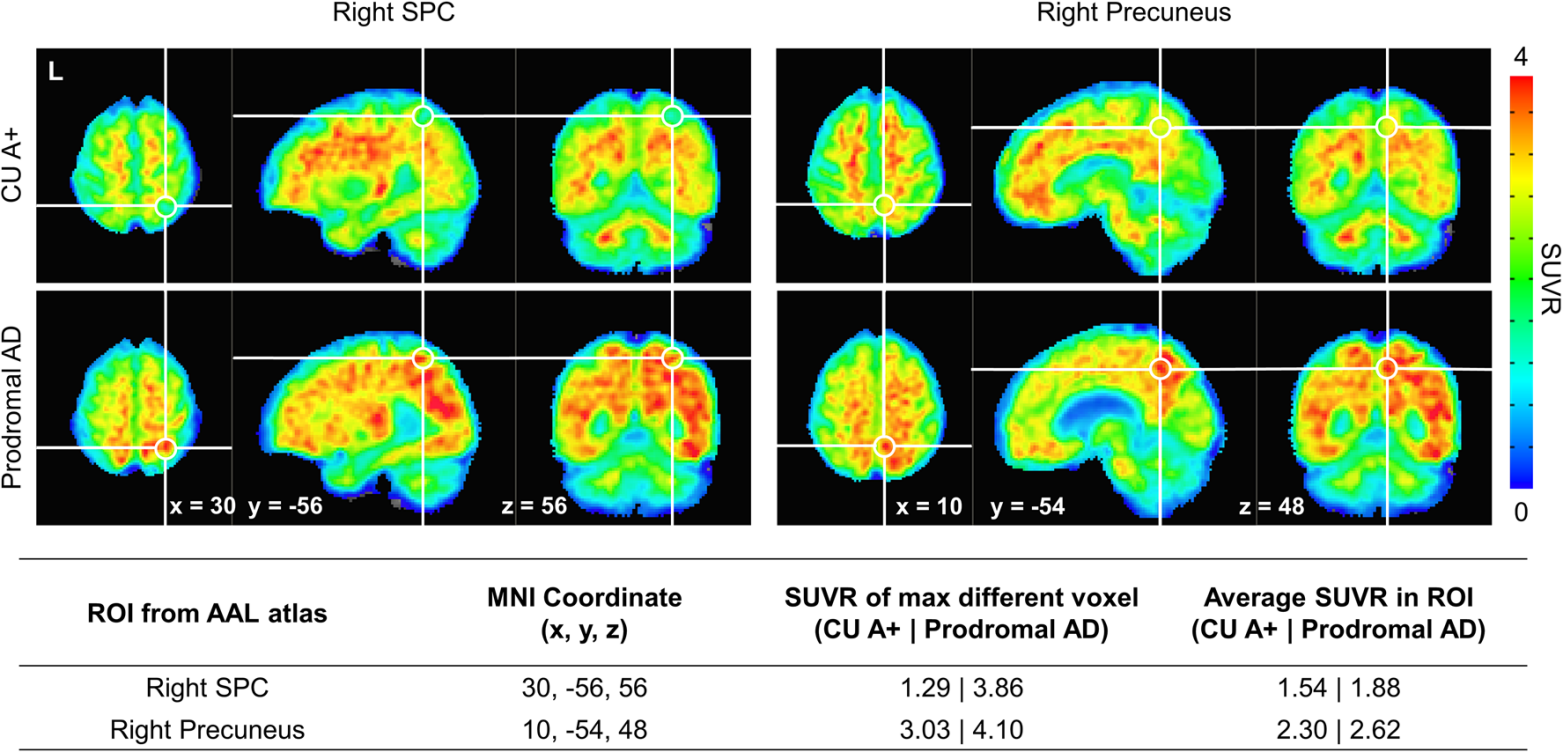
18F-florbetaben brain PET images showing high SUVR in (**A**) right superior parietal cortex (SPC) and (**B**) right precuneus in prodromal Alzheimer’s disease (AD). PET images from one participant with prodromal AD and one age, sex, and education-matched cognitively unimpaired amyloid-positive (CU A+) individual. The white circle indicates the voxel with the largest SUVR difference between CU A+ and prodromal AD. The PET images were coregistered to the individual MRI. The MRI images were subjected to spatial normalization onto the Montreal Neurological Institute (MNI) template using the default unified segmentation methods of Statistical Parametric Mapping (SPM) 12. Using the SPM deformations tool, the computed deformation fields were then applied to the co-registered PET images. The results were normalized PET and MRI images in the MNI space with a voxel size of 2 × 2 × 2 mm^3^. *AAL* Automated anatomical labelling, *CU A*+ cognitively unimpaired amyloid-positive, *ROI* region of interest, *SUVR* standardized uptake value ratio.

In the logistic LASSO regression Model 1, increased SUVRs in the superior parietal cortices and the right precuneus were associated with the likelihood of prodromal AD (Table 3, Fig. 1). In LASSO Model 2, decreased volumes in both the entorhinal cortices, right inferior parietal cortex, both amygdalae, and left hippocampus were associated with prodromal AD diagnosis. In LASSO Model 3, the mean SUVR in the right superior parietal cortex (β = 0.02) and its interaction with regional volume (β = 0.07) were associated with the likelihood of prodromal AD. All selected ROIs in LASSO Model 2 (regional volumes) were retained in LASSO Model 3.

**Table 3:** Selected variables of logistic LASSO regression models for discriminating prodromal Alzheimer’s disease (AD) from cognitively unimpaired amyloid-positive individual.

Regions of interest		Model 1	Model 2	Model 3		
		SUVR	Volume	SUVR	Volume	SUVR volume*
Temporal	L entorhinal cortex		− 0.38		− 0.36	
	R entorhinal cortex		− 0.04		− 0.06	
Parietal	L superior parietal cortex	0.05				
	R inferior parietal cortex		− 0.02		− 0.01	
	R precuneus cortex	0.05				
	R superior parietal cortex	0.05		0.02		0.07
Subcortex	L Amygdala		− 0.12		− 0.12	
	L Hippocampus		− 0.11		− 0.13	
	R Amygdala		− 0.10		− 0.10	
(Intercept)		1.10	1.19	1.19		
Lambda		0.056	0.055	0.055		
Accuracy		0.720	0.745	0.750		
Precision		0.911	0.900	0.914		

*L* left, *LASSO* least absolute shrinkage and selection operator, *R* right, *ROI* region of interest, *SUVR* standardized uptake value ratio.

Model 1 included SUVRs of all sub-ROIs; Model 2 included the volumes of all sub-ROIs, divided by intracranial volume; Model 3 included both the volumes and SUVRs of all sub-ROIs.

*Values show β coefficients in each logistic LASSO models.

**Figure 2:**
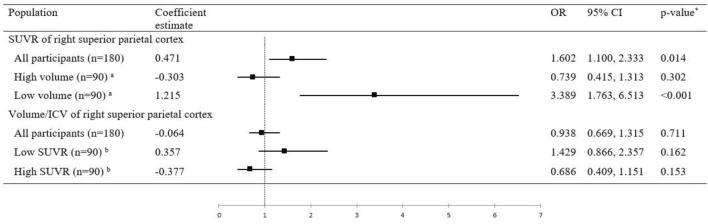
Association of SUVR or volume of right superior parietal cortex with the likelihood of prodromal AD according to subgroups. The black squares and horizontal lines correspond to the ORs and 95% confidence intervals. p-value (*) is a result of logistic regression model in each groups. High volume and low volume groups (a) in SUVR of right superior parietal cortex was grouped by median value of volume in right superior parietal cortex. Low SUVR or High SUVR groups (b) in Volume/ICV of right superior parietal cortex was grouped by median value of SUVR in right superior parietal cortex. *CI* confidence interval, *ICV* intracranial volume, *OR* odds ratio, *SUVR* standardized uptake value ratio.

In the subgroup analyses, increased SUVR in the right superior parietal cortex was associated with the higher likelihood of prodromal AD in the population with a low right superior parietal cortex volume (β = 1.215, 95% CI [0.566, 1.876], p < 0.001; Fig. 2). In the population with a high volume of the right superior parietal cortex, the regional SUVR was not associated with the prodromal AD stage (β =  − 0.303, 95% CI [− 0.879, 0.272], p = 0.302).

## Discussion

In this study, our objective was to explore the regions of the brain that differentiate prodromal AD from CU A+, using regional Aβ SUVR and volume. No ROIs showed a higher SUVR in prodromal AD than in CU A+ group. In contrast, a significant volume reduction in prodromal AD was observed in multiple ROIs including amygdala, hippocampus, entorhinal cortex, insula, parahippocampal gyrus, and inferior temporal and parietal cortices. This suggests that neurodegeneration may be more closely related to the clinical manifestation of AD symptoms in the presence of amyloid positivity, a finding that is also consistent with the amyloid cascade hypothesis^4^. When all ROIs’ SUVR and volume, as well as the interaction between SUVR and volume, were input into a LASSO logistic regression model, SUVR in the right superior parietal cortex and volumes in both entorhinal cortices, right inferior parietal cortex, both amygdalae and left hippocampus showed significant associations with prodromal AD stage. In particular, amyloid deposition and regional atrophy interacted significantly with the right superior parietal cortex. This interaction amplified the association between the amount of amyloid deposition and the prodromal AD stage in groups with lower regional volume in the right superior parietal cortex.

In our study, SUVRs in the superior parietal cortex (Brodmann areas (BAs) 5 and 7) and right precuneus (BA 7 mesial and a small part of BA 31) were associated with prodromal AD in the LASSO regression (Model 1 in Table 3). These regions correspond to mid to late stages in the staging of amyloid accumulation for cognitively unimpaired individuals^7,8^, and to early-to mid-stages of staging throughout the full dementia spectrum^46^. Prospective studies have confirmed these amyloid staging models to reflect the order of amyloid deposition as the disease progresses^12,13^.

The posterior parietal cortex (BAs 5, 7, 39, and 40), to which these regions belong, has been consistently reported in various studies to exhibit structural, functional, and metabolic changes during the early stages of AD^47^. For example, patients with MCI showed an increase in the precuneus, superior parietal cortex, and supramarginal gyrus activation in both memory and non-memory tasks^48–50^. According to the model representing the putative neurobiological mechanisms of parietal vulnerability in the pathogenesis of AD^47^, the parietal lobe possesses a thinner and more susceptible myelin sheath, rendering it highly vulnerable to myelin breakdown^51,52^. This susceptibility leads to the disconnection between the posterior cingulate gyrus/precuneus and the medial temporal lobe area^47,53–56^. Such rupture of axons enhances the deposition of extracellular amyloid^57^, leading to disruptions in glucose metabolism, GM atrophy, and cognitive dysfunction^47^. This model also supports that amyloid accumulation in the regions identified in our study is associated with progression to the symptomatic stage of AD (prodromal AD).

In this study, reduced volumes of the entorhinal cortex, hippocampus, amygdala, and inferior parietal cortex were associated with an increased likelihood of prodromal AD stage. This association persisted even when regional amyloid deposition was incorporated into the LASSO model. These regions are all known to be associated with the conversion from cognitively normal to MCI^58–60^. Furthermore, in the early stages of Braak’s classification of neurofibrillary tangle accumulation (Stages II–IV)^52,61^, these regions have been identified as significant pathological findings in patients with MCI and those showing memory decline^62^. In a recent study on the AD structural progression MRI staging scheme^63^, these regions have also been identified as belonging to the early stages of atrophy. The study suggests that in addition to local tau accumulation, axonal degeneration in remote sites and other limbic-predominant associated proteinopathies may also influence atrophy at these early stages^63^, which seems applicable to the findings of our study as well.

In a model that included amyloid deposition and regional atrophy, the right superior parietal cortex emerged as the only region where amyloid deposition was associated with prodromal AD. In particular, the interaction between regional volume and amyloid deposition in this region was significantly associated with prodromal AD. In a study focusing on prodromal AD and mild AD dementia, this region has been identified as one of the ROIs where the interaction between amyloid positivity and cortical thickness is significantly correlated with the decrease in CDR-sum of boxes^23^. It is recognized that Aβ and tau AD pathologies contribute to cortical thinning and clinical decline^23^, and observations have shown that phosphorylated tau-dependent cortical thinning occurs in amyloid-positive individuals^64,65^. Moreover, some studies have elucidated that Aβ-associated clinical decline manifests only in the presence of elevated phosphorylated tau^66,67^. Therefore, the reduced regional volume in the right superior parietal cortex, which showed a significant association with prodromal AD through interaction with Aβ, is likely due to tau pathology. Additionally, research indicating that global tau-PET signal intensity, rather than amyloid PET, predicts the rate of subsequent atrophy^68^ supports the notion that tau pathology could be a major driver of local neurodegeneration leading to atrophy in the AD brain.

The superior parietal cortex has been traditionally implicated in various cognitive processes^62,69,70^, including sensory integration, visuomotor coordination, higher-order thinking, attention, working memory^71,72^, and episodic memory^73^. As a component of the executive attention network, the superior parietal lobes showed a lower component-related activity in amnestic patients with MCI compared to normal controls^74^. In patients at risk of developing AD, the activity of the superior parietal cortex changes during executive attention-related tasks^48^. A recent study suggests that a connection within the default mode network, particularly between the right superior parietal lobule and the precuneus, may be related to memory capabilities, as indicated by its association with the CDR memory subscale^75^. These functional characteristics of the superior parietal cortex also support the possibility that this region reflects the symptom expression in AD continuums, specifically prodromal AD.

A recent study^76^ that observed the asymmetry of amyloid deposition in preclinical AD showed a tendency to leftward lateralization in the preclinical phase, which transitioned to more symmetric accumulation as the disease progressed to the symptomatic stage. Given this background, it is plausible that the more pronounced amyloid deposition in the right-sided regions, including the right superior parietal cortex, when comparing preclinical and prodromal AD, could be attributed to the change from left-sided deposition in the preclinical phase to a more concentrated deposition in the right-sided regions in prodromal AD.

In our study, we implemented a 1:3 matching for age, education, and sex, improving the robustness of our results and effectively minimizing potential confounding variables. Furthermore, by simultaneously examining regional volume and amyloid deposition at the whole-brain level, we were able to identify significant regions and delve into their intricate interactions. Using the LASSO technique in our logistic regression analyses allowed for the selection of the most pertinent variables, thus reducing potential biases from multicollinearity and further strengthening the robustness of our findings. However, the limitations of our study warrant further consideration. First, this study was a cross-sectional study, we were unable to infer causality or direction of effect between Aβ and regional atrophy. Second, we did not examine the potential for interactions between atrophy in one ROI and regional atrophy in a different ROI to reflect progression to prodromal AD. Third, we did not match other potential confounders, such as the APOE genotype or various lifestyle factors, both of which could influence brain amyloid deposition, volume, and advanced clinical stages. Future research should ensure a sufficient sample size matched not only for age, sex, and education but also for ApoE4 status, to facilitate separate analyses based on the presence or absence of the ApoE4 allele. Finally, due to the absence of tau PET data, we were unable to directly investigate the impact of tau pathology on cognitive stage in AD.

This study investigated the impact of amyloid deposition, atrophy, and their interaction in regions associated with the prodromal AD stage compared to CU A+ in the AD continuum. Regional brain atrophy may be more closely associated with the early symptomatic stages of AD than with amyloid deposition, which is consistent with the results of previous studies. The accumulation of amyloid in the right superior parietal cortex, especially with a lower regional volume, could have a substantial association with the symptomatic phase of AD. These findings can be used to identify individuals with symptomatic AD among amyloid-positive individuals using PET and MRI data and can help to understand the pathological processes underlying the progression of AD.

### Supplementary Information

Supplementary Information.

## Data Availability

The anonymized data used and/or analyzed in this report are available from the corresponding authors upon reasonable requests.
