# Supplementary material for: Exploration of neuroanatomical characteristics to differentiate prodromal Alzheimer’s disease from cognitively unimpaired amyloid-positive individuals
Source: Sci Rep. 2024 May 2;14:10083. doi: 10.1038/s41598-024-60843-8 (PMC11066072; doi:10.1038/s41598-024-60843-8)

Supplementary Table S1. Comparisons of regional SUVR and volume between cognitively unimpaired amyloid-positive individual (CU A+) and prodromal Alzheimer’s disease in each region of interest of Desikan-Killiany-Tourville Atlas

|  | SUVR | | |  | Regional volume / ICV (%) | | |
| --- | --- | --- | --- | --- | --- | --- | --- |
| Regions of interest | CU A+ | Prodromal AD | p-value |  | CU A+ | Prodromal AD | p-value |
| Frontal lobe |  |  |  |  |  |  |  |
| Left caudal middle frontal gyrus | 1.30 ± 0.26 | 1.31 ± 0.26 | 0.808 |  | 0.34 ± 0.04 | 0.34 ± 0.05 | 0.481 |
| Left frontal pole | 1.44 ± 0.36 | 1.49 ± 0.36 | 0.465 |  | 0.06 ± 0.01 | 0.06 ± 0.01 | 0.662 |
| Left lateral orbitofrontal cortex | 1.49 ± 0.31 | 1.44 ± 0.29 | 0.486 |  | 0.42 ± 0.04 | 0.41 ± 0.04 | 0.279 |
| Left medial orbitofrontal cortex | 1.43 ± 0.31 | 1.43 ± 0.31 | 1.000 |  | 0.30 ± 0.03 | 0.29 ± 0.03 | 0.188 |
| Left pars opercularis | 1.34 ± 0.23 | 1.36 ± 0.27 | 0.746 |  | 0.24 ± 0.03 | 0.24 ± 0.04 | 0.696 |
| Left pars orbitalis | 1.41 ± 0.29 | 1.43 ± 0.30 | 0.722 |  | 0.12 ± 0.02 | 0.12 ± 0.02 | 0.755 |
| Left pars triangularis | 1.43 ± 0.26 | 1.44 ± 0.29 | 0.764 |  | 0.20 ± 0.03 | 0.20 ± 0.03 | 0.732 |
| Left rostral middle frontal gyrus | 1.51 ± 0.32 | 1.54 ± 0.29 | 0.554 |  | 0.83 ± 0.09 | 0.80 ± 0.08 | 0.023 ^a^ |
| Left superior frontal gyrus | 1.29 ± 0.27 | 1.31 ± 0.25 | 0.704 |  | 1.25 ± 0.13 | 1.21 ± 0.11 | 0.031 ^a^ |
| Left precentral gyrus | 1.03 ± 0.22 | 1.06 ± 0.22 | 0.435 |  | 0.80 ± 0.08 | 0.78 ± 0.09 | 0.213 |
| Right caudal middle frontal gyrus | 1.30 ± 0.27 | 1.33 ± 0.26 | 0.584 |  | 0.32 ± 0.04 | 0.32 ± 0.05 | 0.593 |
| Right frontal pole | 1.45 ± 0.35 | 1.49 ± 0.35 | 0.438 |  | 0.07 ± 0.01 | 0.07 ± 0.01 | 0.837 |
| Right lateral orbitofrontal cortex | 1.41 ± 0.27 | 1.43 ± 0.29 | 0.717 |  | 0.41 ± 0.04 | 0.40 ± 0.04 | 0.049 ^a^ |
| Right medial orbitofrontal cortex | 1.48 ± 0.29 | 1.49 ± 0.32 | 0.789 |  | 0.31 ± 0.03 | 0.31 ± 0.03 | 0.898 |
| Right pars opercularis | 1.39 ± 0.23 | 1.41 ± 0.29 | 0.682 |  | 0.21 ± 0.03 | 0.21 ± 0.03 | 0.710 |
| Right pars orbitalis | 1.43 ± 0.25 | 1.47 ± 0.30 | 0.330 |  | 0.15 ± 0.02 | 0.14 ± 0.02 | 0.037 ^a^ |
| Right pars triangularis | 1.44 ± 0.25 | 1.49 ± 0.28 | 0.329 |  | 0.22 ± 0.03 | 0.23 ± 0.03 | 0.368 |
| Right rostral middle frontal gyrus | 1.52 ± 0.34 | 1.57 ± 0.28 | 0.323 |  | 0.83 ± 0.09 | 0.81 ± 0.08 | 0.046 ^a^ |
| Right superior frontal gyrus | 1.28 ± 0.27 | 1.32 ± 0.24 | 0.413 |  | 1.17 ± 0.12 | 1.13 ± 0.10 | 0.075 |
| Right precentral gyrus | 1.05 ± 0.22 | 1.07 ± 0.21 | 0.578 |  | 0.79 ± 0.09 | 0.77 ± 0.08 | 0.085 |
| Limbic system |  |  |  |  |  |  |  |
| Left caudal anterior cingulate cortex | 1.34 ± 0.25 | 1.35 ± 0.25 | 0.709 |  | 0.09 ± 0.02 | 0.09 ± 0.02 | 0.225 |
| Left rostral anterior cingulate cortex | 1.46 ± 0.24 | 1.45 ± 0.30 | 0.788 |  | 0.14 ± 0.02 | 0.14 ± 0.02 | 0.111 |
| Left isthmus cingulate cortex | 1.38 ± 0.26 | 1.43 ± 0.26 | 0.277 |  | 0.15 ± 0.02 | 0.14 ± 0.02 | 0.064 |
| Left insula | 1.04 ± 0.19 | 1.05 ± 0.22 | 0.635 |  | 0.42 ± 0.04 | 0.41 ± 0.04 | 0.028 ^a^ |
| Left parahippocampal gyrus | 0.94 ± 0.18 | 0.94 ± 0.21 | 0.945 |  | 0.11 ± 0.02 | 0.10 ± 0.02 | 0.014 ^a^ |
| Left posterior cingulate cortex | 1.41 ± 0.30 | 1.47 ± 0.27 | 0.209 |  | 0.18 ± 0.03 | 0.17 ± 0.03 | 0.014 ^a^ |
| Right caudal anterior cingulate cortex | 1.32 ± 0.25 | 1.35 ± 0.25 | 0.442 |  | 0.12 ± 0.03 | 0.11 ± 0.03 | 0.141 |
| Right rostral anterior cingulate cortex | 1.45 ± 0.24 | 1.47 ± 0.28 | 0.633 |  | 0.11 ± 0.03 | 0.11 ± 0.02 | 0.231 |
| Right isthmus cingulate cortex | 1.40 ± 0.23 | 1.46 ± 0.27 | 0.182 |  | 0.14 ± 0.02 | 0.14 ± 0.02 | 0.094 |
| Right insula | 1.04 ± 0.18 | 1.06 ± 0.22 | 0.508 |  | 0.43 ± 0.04 | 0.41 ± 0.04 | 0.003 ^a,b^ |
| Right parahippocampal gyrus | 0.96 ± 0.19 | 0.98 ± 0.23 | 0.491 |  | 0.11 ± 0.02 | 0.10 ± 0.02 | 0.002 ^a,b^ |
| Right posterior cingulate cortex | 1.39 ± 0.28 | 1.46 ± 0.26 | 0.101 |  | 0.18 ± 0.03 | 0.18 ± 0.03 | 0.565 |
| Temporal lobe |  |  |  |  |  |  |  |
| Left bankssts | 1.69 ± 0.34 | 1.75 ± 0.39 | 0.346 |  | 0.13 ± 0.02 | 0.12 ± 0.02 | 0.175 |
| Left entorhinal cortex | 0.67 ± 0.18 | 0.64 ± 0.23 | 0.374 |  | 0.13 ± 0.02 | 0.10 ± 0.03 | <0.001 ^a,b^ |
| Left inferior temporal gyrus | 1.34 ± 0.29 | 1.34 ± 0.31 | 0.984 |  | 0.64 ± 0.08 | 0.60 ± 0.08 | 0.009 ^a,b^ |
| Left middle temporal gyrus | 1.38 ± 0.27 | 1.38 ± 0.30 | 0.913 |  | 0.60 ± 0.08 | 0.59 ± 0.07 | 0.187 |
| Left superior temporal gyrus | 1.24 ± 0.28 | 1.29 ± 0.33 | 0.331 |  | 0.68 ± 0.07 | 0.66 ± 0.08 | 0.055 |
| Left temporal pole | 0.90 ± 0.25 | 0.90 ± 0.27 | 0.907 |  | 0.15 ± 0.02 | 0.15 ± 0.03 | 0.167 |
| Left transverse temporal cortex | 1.25 ± 0.41 | 1.33 ± 0.42 | 0.243 |  | 0.06 ± 0.01 | 0.06 ± 0.01 | 0.846 |
| Right bankssts | 1.68 ± 0.32 | 1.74 ± 0.37 | 0.326 |  | 0.12 ± 0.02 | 0.11 ± 0.02 | 0.389 |
| Right entorhinal cortex | 0.67 ± 0.17 | 0.67 ± 0.21 | 0.939 |  | 0.12 ± 0.02 | 0.11 ± 0.02 | <0.001 ^a,b^ |
| Right inferior temporal gyrus | 1.34 ± 0.28 | 1.34 ± 0.29 | 0.928 |  | 0.60 ± 0.08 | 0.57 ± 0.07 | 0.047 ^a^ |
| Right middle temporal gyrus | 1.38 ± 0.26 | 1.39 ± 0.28 | 0.858 |  | 0.65 ± 0.07 | 0.63 ± 0.07 | 0.122 |
| Right superior temporal gyrus | 1.25 ± 0.25 | 1.29 ± 0.30 | 0.423 |  | 0.64 ± 0.06 | 0.62 ± 0.07 | 0.146 |
| Right temporal pole | 0.91 ± 0.23 | 0.90 ± 0.24 | 0.904 |  | 0.16 ± 0.03 | 0.15 ± 0.03 | 0.355 |
| Right transverse temporal cortex | 1.19 ± 0.36 | 1.27 ± 0.41 | 0.235 |  | 0.05 ± 0.01 | 0.05 ± 0.01 | 0.054 |
| Parietal lobe |  |  |  |  |  |  |  |
| Left inferior parietal cortex | 1.50 ± 0.28 | 1.56 ± 0.33 | 0.263 |  | 0.67 ± 0.09 | 0.65 ± 0.09 | 0.088 |
| Left paracentral lobule | 1.08 ± 0.30 | 1.17 ± 0.27 | 0.061 |  | 0.22 ± 0.02 | 0.21 ± 0.03 | 0.048 ^a^ |
| Left postcentral gyrus | 0.99 ± 0.29 | 1.08 ± 0.30 | 0.076 |  | 0.54 ± 0.07 | 0.53 ± 0.06 | 0.344 |
| Left precuneus cortex | 1.55 ± 0.33 | 1.64 ± 0.32 | 0.108 |  | 0.52 ± 0.07 | 0.52 ± 0.06 | 0.571 |
| Left superior parietal cortex | 1.25 ± 0.30 | 1.39 ± 0.34 | 0.015 ^a,c^ |  | 0.73 ± 0.11 | 0.73 ± 0.10 | 0.732 |
| Left supramarginal gyrus | 1.34 ± 0.25 | 1.42 ± 0.30 | 0.121 |  | 0.63 ± 0.10 | 0.61 ± 0.09 | 0.224 |
| Right inferior parietal cortex | 1.50 ± 0.26 | 1.57 ± 0.30 | 0.138 |  | 0.81 ± 0.09 | 0.77 ± 0.10 | 0.006 ^a,b^ |
| Right paracentral lobule | 1.12 ± 0.31 | 1.19 ± 0.26 | 0.153 |  | 0.23 ± 0.03 | 0.23 ± 0.03 | 0.391 |
| Right postcentral gyrus | 1.01 ± 0.28 | 1.08 ± 0.30 | 0.173 |  | 0.53 ± 0.07 | 0.51 ± 0.06 | 0.216 |
| Right precuneus cortex | 1.50 ± 0.31 | 1.64 ± 0.33 | 0.014 ^a,c^ |  | 0.55 ± 0.07 | 0.54 ± 0.05 | 0.492 |
| Right superior parietal cortex | 1.22 ± 0.28 | 1.36 ± 0.33 | 0.012 ^a,c^ |  | 0.72 ± 0.10 | 0.72 ± 0.09 | 0.712 |
| Right supramarginal gyrus | 1.30 ± 0.25 | 1.41 ± 0.30 | 0.039 ^a,c^ |  | 0.57 ± 0.09 | 0.54 ± 0.07 | 0.120 |
| Occipital lobe |  |  |  |  |  |  |  |
| Left pericalcarine cortex | 1.12 ± 0.59 | 1.15 ± 0.58 | 0.755 |  | 0.12 ± 0.02 | 0.12 ± 0.02 | 0.975 |
| Left fusiform gyrus | 1.25 ± 0.26 | 1.23 ± 0.28 | 0.686 |  | 0.53 ± 0.07 | 0.51 ± 0.07 | 0.121 |
| Left cuneus cortex | 1.09 ± 0.45 | 1.07 ± 0.47 | 0.799 |  | 0.17 ± 0.03 | 0.17 ± 0.02 | 0.804 |
| Left lateral occipital cortex | 1.32 ± 0.37 | 1.35 ± 0.40 | 0.730 |  | 0.66 ± 0.08 | 0.64 ± 0.09 | 0.143 |
| Left lingual gyrus | 1.01 ± 0.36 | 0.99 ± 0.37 | 0.823 |  | 0.36 ± 0.05 | 0.35 ± 0.05 | 0.173 |
| Right pericalcarine cortex | 1.19 ± 0.59 | 1.17 ± 0.62 | 0.796 |  | 0.13 ± 0.02 | 0.13 ± 0.02 | 0.862 |
| Right fusiform gyrus | 1.24 ± 0.27 | 1.22 ± 0.28 | 0.703 |  | 0.52 ± 0.09 | 0.49 ± 0.06 | 0.108 |
| Right cuneus cortex | 1.11 ± 0.44 | 1.08 ± 0.50 | 0.684 |  | 0.18 ± 0.03 | 0.18 ± 0.02 | 0.217 |
| Right lateral occipital | 1.40 ± 0.37 | 1.36 ± 0.41 | 0.577 |  | 0.66 ± 0.09 | 0.63 ± 0.08 | 0.088 |
| Right lingual gyrus | 1.01 ± 0.38 | 0.99 ± 0.40 | 0.702 |  | 0.38 ± 0.05 | 0.37 ± 0.06 | 0.226 |
| Subcortex |  |  |  |  |  |  |  |
| Left accumbens | 1.48 ± 0.29 | 1.49 ± 0.32 | 0.789 |  | 0.03 ± 0.01 | 0.02 ± 0.01 | 0.175 |
| Left amygdala | 1.30 ± 0.27 | 1.33 ± 0.26 | 0.584 |  | 0.08 ± 0.01 | 0.07 ± 0.01 | <0.001 ^a,b^ |
| Left caudate | 1.41 ± 0.29 | 1.43 ± 0.30 | 0.722 |  | 0.22 ± 0.03 | 0.21 ± 0.03 | 0.370 |
| Left hippocampus | 1.44 ± 0.36 | 1.49 ± 0.36 | 0.465 |  | 0.22 ± 0.03 | 0.20 ± 0.03 | <0.001 ^a,b^ |
| Left pallidum | 1.51 ± 0.32 | 1.54 ± 0.29 | 0.554 |  | 0.11 ± 0.02 | 0.11 ± 0.02 | 0.692 |
| Left putamen | 1.43 ± 0.26 | 1.44 ± 0.29 | 0.764 |  | 0.27 ± 0.04 | 0.27 ± 0.03 | 0.351 |
| Right accumbens | 1.40 ± 0.23 | 1.46 ± 0.27 | 0.182 |  | 0.03 ± 0.01 | 0.03 ± 0.00 | 0.177 |
| Right amygdala | 1.32 ± 0.25 | 1.35 ± 0.25 | 0.442 |  | 0.09 ± 0.01 | 0.09 ± 0.01 | <0.001 ^a,b^ |
| Right caudate | 1.34 ± 0.25 | 1.35 ± 0.25 | 0.709 |  | 0.22 ± 0.03 | 0.21 ± 0.03 | 0.215 |
| Right hippocampus | 1.46 ± 0.24 | 1.45 ± 0.30 | 0.788 |  | 0.23 ± 0.04 | 0.21 ± 0.03 | <0.001 ^a,b^ |
| Right pallidum | 1.41 ± 0.30 | 1.47 ± 0.27 | 0.209 |  | 0.11 ± 0.02 | 0.11 ± 0.02 | 0.809 |
| Right putamen | 1.38 ± 0.26 | 1.43 ± 0.26 | 0.277 |  | 0.28 ± 0.03 | 0.27 ± 0.03 | 0.238 |

Standardized data are presented as mean ± SD.

^a^ p<0.05 from independent t-test between CU A+ and prodromal AD

^b^ False Discovery Rate adjusted p value <0.1 from independent t-test between CU A+ and prodromal AD; no regions of interest were significant at FDR p <0.05 level

^c^ p<0.05 from analysis of covariance (ANCOVA) between CU A+ and prodromal AD, adjusted by volume/ICV of each ROI

Abbreviations: AD, Alzheimer’s disease; Bankssts, banks of the superior temporal sulcus; CU A+; cognitively unimpaired amyloid positive individual, ICV, intracranial volume; ROI, region of interest; SD, standard deviation; SUVR, standardized uptake value ratio;

Supplementary Figure S2. Voxel-based analysis between the cognitively unimpaired amyloid-positive individual (CU A+, preclinical Alzheimer’s disease [AD]) and prodromal AD groups (using the 3dClustSim software within the AFNI suite (https://afni.nimh.nih.gov/) to determine the significance of clusters)

1. A voxel-based analysis of the SUVR between the cognitively unimpaired amyloid-positive individual (CU A+, preclinical Alzheimer’s disease [AD]) and prodromal AD groups. No clusters with significant differences between the groups were found at an uncorrected p-value of 0.001.
2. Voxel-based morphometry between the two groups, the peak labels (Automated Anatomical Labeling [AAL] atlas) of clusters larger than 100 at an uncorrected p < 0.001 level were the left fusiform gyrus, left parahippocampal gyrus, right postcentral gyrus, right middle temporal gyrus, left middle cingulate cortex, left precuneus, and left insula (Panel **A**, CU A+ > prodromal AD group). Upon correction for multiple comparisons at uncorrected p < 0.005 and Family-Wise Error rate (FWE) < 0.05 (cluster size > 4220 voxels), left fusiform gyrus (peak level, Panel **B**), left parahippocampal gyrus, left amygdala, and left insula were identified. Panel **C**: Clusters according to the z-coordinate. Shown at intervals of 2 from z = -46 to -12.


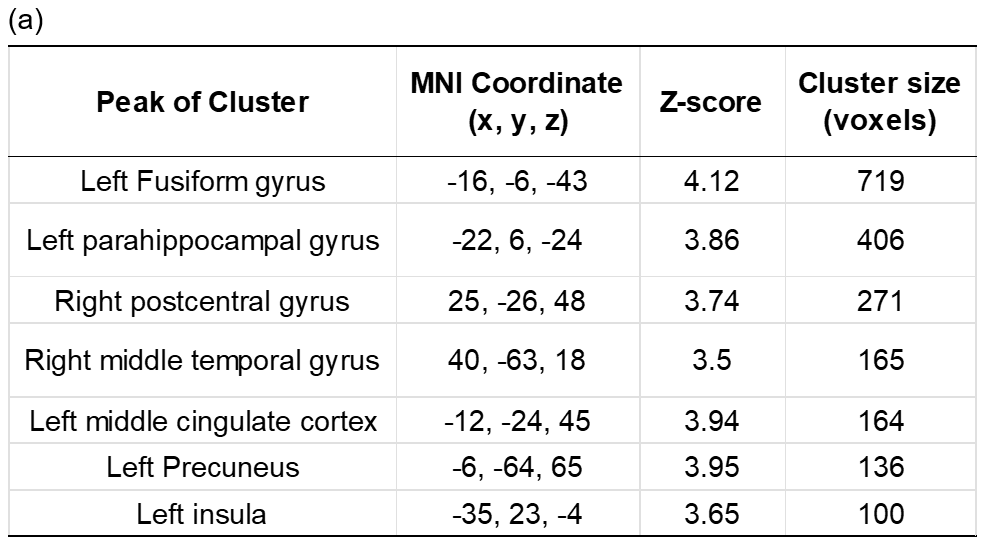


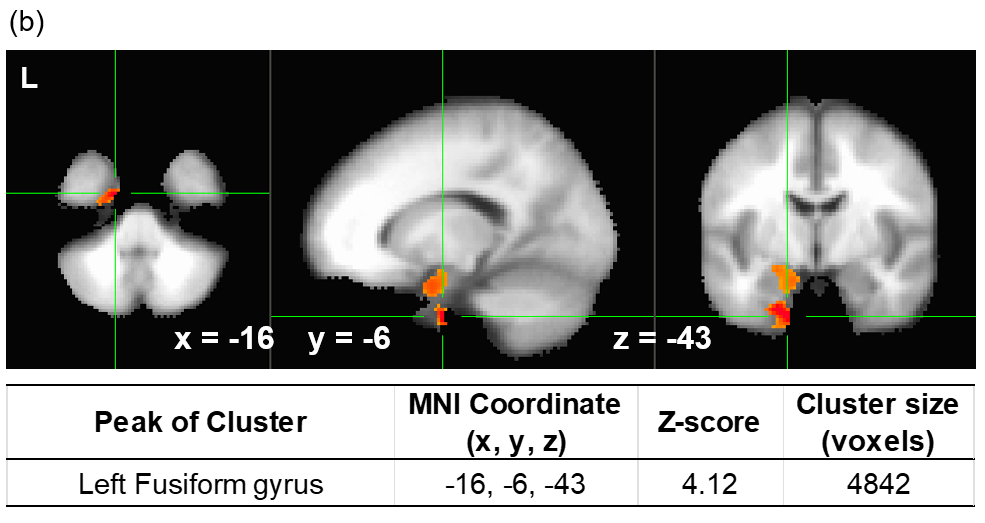


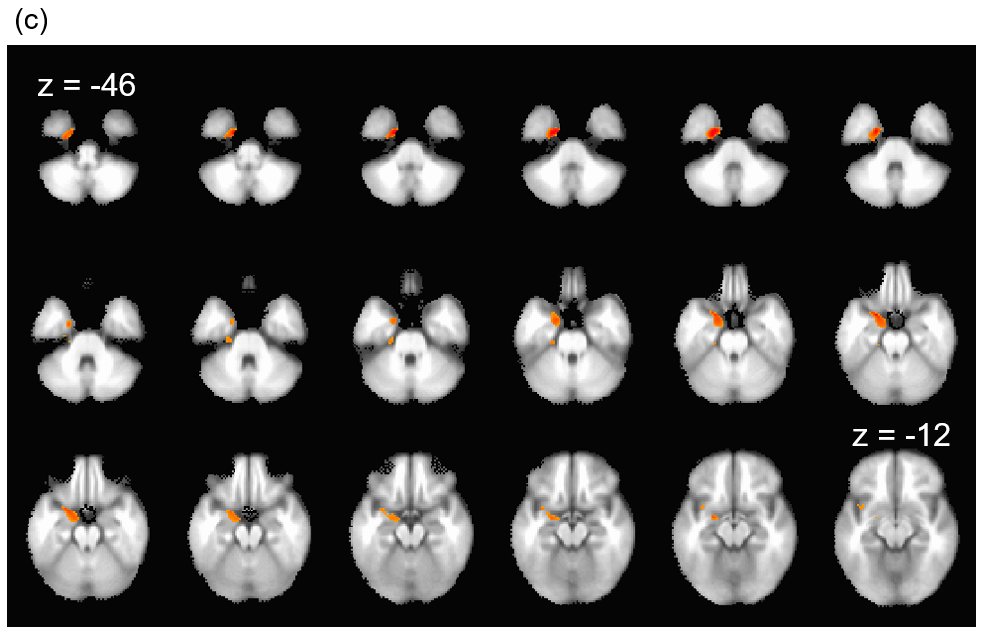

Supplement: Supplementary file 1 — Supplementary Information. [file 41598_2024_60843_MOESM1_ESM.docx]
